# Editorial: Craniofacial neuroscience

**DOI:** 10.3389/fneur.2025.1621802

**Published:** 2025-07-03

**Authors:** Alyssa Huff, Kei A. Katsura, Teresa Pitts

**Affiliations:** ^1^Seattle Children's Research Institute, Norcliffe Foundation Center for Integrative Brain Research, Seattle, WA, United States; ^2^Department of Human Genetics, Children's Hospital of Philadelphia, Philadelphia, PA, United States; ^3^ITMAT School of Medicine, University of Pennsylvania, Philadelphia, PA, United States; ^4^Department of Orofacial Sciences, University of California, San Francisco, San Francisco, CA, United States; ^5^Department of Speech, Language and Hearing Sciences, Dalton Cardiovascular Center Investigator, University of Missouri, Columbia, MO, United States

**Keywords:** dysphagia, breathing, pain, expression, cranial nerves

The 12 cranial nerves (CNs) are essential for life-sustaining behaviors (feeding, swallowing, coughing, chewing, and breathing) and are vital to communication (sight, speech, and facial expressions). These functions require precise and coordinated control of the nerves, muscles, and hard tissues of the craniofacial complex, which are influenced by sensory input. Craniofacial impairments, abnormalities, and disorders can result from genetic conditions, neurodegeneration, neurotrauma, and/or cranial nerve damage. They affect people of all age groups, sexes, and ethnicities. These impairments affect quality of life through decreased oral hygiene, psychosocial anxiety, chronic pain, speech and sight impairments, developmental delays, dysphagia, and/or dystussia (coughing reflex). These can lead to malnutrition, failure to thrive, and sometimes premature death. Scientific investigations of these impairments are often overlooked due to their complex nature, which spans across multiple fields and specialties such as genetics, physiology, neuroscience, critical care, speech and language pathology, physical and occupational therapy, and dentistry. In this editorial, we will briefly review the impact of the CNs on swallowing, breathing, pain, and expression.

Upper airway dysfunction continues to be detailed in a number of neurodegenerative and neurotraumatic diseases. Song et al. reported that the prevalence of post-stroke dysphagia is 47% with associated risk factors such as hypertension, previous stroke, and atrial fibrillation. The authors also reported the persistence of dysphagia at discharge and 1 month post to be 75% and 51%, respectively. Martinez-Peña et al. also reported a high prevalence of dysphagia in older individuals with mild cognitive impairment and dementia. This suggests that early identification and intervention are key to preventing serious health outcomes. Liu et al. reported an association between lower income levels and increased oropharyngeal dysphagia, highlighting the complex challenges of healthcare accessibility, nutritional adequacy and stress. The authors also called for a sex-specific approach to early intervention in the aging population.

Preclinical animal models are investigating several potential mechanisms of dysphagia and upper airway dysfunction. Motoneuron (MN) death associated with amyotrophic lateral sclerosis (ALS) disrupts orolingual and aerodigestive behaviors such as speech and swallowing, in addition to ventilatory and non-ventilatory behaviors, breathing, and coughing, respectively. These deficits also result in an increased risk of airway protection and aspiration pneumonia. Using a mouse model of ALS, Fogarty et al. investigated the timeline of size-dependent XII MN loss and tongue innervation in SOD1 mice. They observed a significant reduction in larger XII MNs at the mid- and end-stage of the disease with no difference at the pre-symptomatic or onset ages. Specific disruption to neuromuscular junctions in the tongue did not occur until the end-stage, leading the authors to conclude that denervation of neuromuscular junctions in the tongue may be a consequence, not a cause, of MN deficits in SOD1 mice. Meanwhile Keilholz et al. used a different rodent model to induce XII MN death and investigated the use of a strength endurance tongue exercise program as a potential therapy for patients with motoneuron disease and ALS. They found that tongue exercise mitigated airflow deficits and preserved the upper airway and ultrafine structures in the tongue, suggesting that a high-repetition, low-endurance tongue exercise program could be an effective therapy to maintain upper airway patency.

Patients with Down Syndrome experience dysphagia across all three swallowing phases: oral, pharyngeal, and esophageal. This is likely due to disordered tongue function, uncoordinated swallowing and breathing, and esophageal dysmotility. Glass et al. utilized clinical techniques with a mouse model of Down Syndrome (Ts65Dn). They found that adult Ts65Dn mice have significantly slower swallow rates and longer time intervals between consecutive swallows. Adult Ts65Dn mice also have slower rates of developing tongue force and a more rapid onset of tongue muscle fatigue. The authors concluded that heightened susceptibility to tongue muscle fatigue could contribute to an increased duration of the oral phase and decreased efficacy of deglutition (swallowing).

The use of opioids, specifically morphine and remifentanil, has been associated with aspiration and swallowing dysfunction. However, the influence of codeine, the most abused opioid drug worldwide, on swallowing is unknown. Bolser et al. reported that intravenous codeine induced spontaneous swallowing in vagally intact and vagally denervated cats, suggesting that the swallow-promoting actions of this drug do not require sensory feedback from the vagus nerve to occur. Although there was an increase in the amplitude of upper airway muscles during water-induced swallows, swallow frequency did not change. This finding continues to support the concept that swallow frequency and swallow amplitude are independently regulated.

While swallowing is thought to be predominantly controlled by the brainstem, there is strong evidence that the spinal cord also plays an important role. Kitamura et al. utilized Gaussian frequency stimulation applied to the skin surface of the back over the ribs in a rodent model to activate sensory spinal pathways as a possible therapy to improve swallowing activity. Stimulation at the T9-T10 level significantly increased swallowing-related muscle amplitudes of the mylohyoid, thyroarytenoid, and thyropharyngeus. The mylohyoid is a laryngeal elevator muscle, while the thyropharyngeus is a pharyngeal constrictor muscle that is crucial for bolus transfer during swallowing. The thyroarytenoid is a laryngeal adductor muscle that activates during swallowing to close the airway and prevent food or liquid from entering. Hashimoto et al. sought to develop a new dysphagia model with reduced pharyngeal constriction during the pharyngeal phase of swallowing in guinea pigs. Denervation of the pharyngeal branch of the vagus nerve significantly impacted the expiratory and swallowing-related activity of the thyropharyngeus and disrupted swallow function 1 month after injury. This new experimental model could provide insight into the development of dysphagia therapies and the mechanisms associated with cranial nerve injury, reinnervation, and regeneration. The motor neurons that regulate these pharyngeal and laryngeal muscles are located within the nucleus ambiguus. Fogarty characterized the dendritic morphology of MNs and non-MNs within the compact, semi-compact, and loose formations of the nucleus ambiguus in the brainstem. These findings provide valuable insight into the inter-network connections of these neurons, which must coordinate for swallowing to function properly.

Beyond ingestion, the trigeminal nerve is responsible for both sensory and motor functions of the face, by providing motor innervation to the muscles of mastication and sensory feedback from the jaws and teeth. The mesencephalic trigeminal nucleus, a key portion of the CN V, is activated during bruxism, which is the repetitive clenching or grinding of the teeth. This behavior can occur while a person is awake or asleep and affects up to 30% of the world population. Uchima Koecklin et al. described the current understanding of the brain regions and neurotransmitters involved in bruxism, along with the associated psychological traits and clinical implications. The authors encourage continued animal and human studies on bruxism, specifically of the trigeminal system, to improve treatment approaches and understanding of this multifactorial condition.

In dental procedures, damage to branches of the trigeminal nerve (inferior alveolar nerve and lingual nerve) is often the major complication following lower jaw surgeries and third molar extractions and can lead to temporary and/or permanent numbness in the lower lip and tongue. Facial Palsy refers to weakness or paralysis of the facial muscles caused by damage to the facial nerve. Facial synkinesis is a complication that can develop after facial palsy, which refers to the involuntary, simultaneous movement of the facial muscles. These conditions can affect facial expressions, eating, drinking, speech, and eye closure, hindering daily activities and quality of life. Machetanz et al. drew attention to the lack of specialized treatment options for facial palsy and the fact that the vast number of specialists required for its treatment contributes to treatment satisfaction and better quality of life. Di Stadio et al. presented the idea of pairing physical facial nerve rehabilitation with early lower eyelid surgery to improve or prevent synkinesis. They reported that patients who underwent eyelid surgery along with physical facial nerve rehabilitation experienced faster and better recovery of facial movements and had no synkinesis even 24 months after surgery. Conversely, 37% of patients who did not undergo surgery and only participated in physical facial nerve rehabilitation developed synkinesis.

While the primary focus of craniofacial neuroscience research is to support our understanding of human populations, this knowledge is also extremely valuable in the rehabilitation of other species, including harbor seals. Thousands of infant harbor seals have been admitted to the Vancouver Aquarium's Marine Mammal Rescue Center and other rehabilitation centers worldwide. The primary cause of death in seal pups is malnutrition, and in adults it is pneumonia. Skoretz et al. described the novel ability to use clinical Videofluoroscopic Swallow Studies (VFSs) in seal pups to identify four distinct swallowing phases, which vastly expands our understanding of airway protection in independently feeding seals.

This Research Topic of articles draws attention to the wide variety of specialties associated with the 12 cranial nerves and their role in many different behaviors and conditions. Continued collaboration between basic and clinical scientists is necessary to improve our understanding of craniofacial neuroscience.

